# Development of a *Nasonia vitripennis* outbred laboratory population for genetic analysis

**DOI:** 10.1111/1755-0998.12201

**Published:** 2013-12-12

**Authors:** Louis van de Zande, Steven Ferber, Ammerins de Haan, Leo W Beukeboom, Joost van Heerwaarden, Bart A Pannebakker

**Affiliations:** * Evolutionary Genetics, Centre for Ecological and Evolutionary Studies, University of Groningen9700 CC, Groningen, the Netherlands; † Biometris, Wageningen University6708 PB, Wageningen, the Netherlands; ‡ Laboratory of Genetics, Wageningen University6708 PB, Wageningen, the Netherlands

**Keywords:** effective population size, genetic variation, laboratory strain, parasitoid wasp, pooled resequencing, single-nucleotide polymorphism (SNP)

## Abstract

The parasitoid wasp genus *Nasonia* has rapidly become a genetic model system for developmental and evolutionary biology. The release of its genome sequence led to the development of high-resolution genomic tools, for both interspecific and intraspecific research, which has resulted in great advances in understanding *Nasonia* biology. To further advance the utility of *Nasonia vitripennis* as a genetic model system and to be able to fully exploit the advantages of its fully sequenced and annotated genome, we developed a genetically variable and well-characterized experimental population. In this study, we describe the establishment of the genetically diverse HVRx laboratory population from strains collected from the field in the Netherlands. We established a maintenance method that retains genetic variation over generations of culturing in the laboratory. As a characterization of its genetic composition, we provide data on the standing genetic variation and estimate the effective population size (*N*_e_) by microsatellite analysis. A genome-wide description of polymorphism is provided through pooled resequencing, which yielded 417 331 high-quality SNPs spanning all five *Nasonia* chromosomes. The HVRx population and its characterization are freely available as a community resource for investigators seeking to elucidate the genetic basis of complex trait variation using the *Nasonia* model system.

## Introduction

The parasitoid wasp genus *Nasonia* has rapidly become a genetic model system for developmental and evolutionary biology (Desplan & Beukeboom 2003; Werren & Loehlin 2009; Brown 2010; Godfray 2010; Muers 2010). As a genetic model, it has inherent advantages that rival even the current model insect systems with a much longer history and tradition. Its haplodiploid reproduction offers advantages of haploid genetics (e.g. lack of genetic dominance) in a complex eukaryotic system (Pultz *et al.* 2000). Several other important characteristics (easy husbandry, visible mutation markers, four interfertile species, short-generation time and large family sizes, long-term storage after diapause induction) have laid the foundations for the large-scale study of *Nasonia*. However, the most important step in the development of *Nasonia* as a genetic model has been the availability of its genome sequence (Werren *et al.* 2010), which largely expanded the understanding of basic biological processes in this species complex.

Of all the members within the *Nasonia* genus, *Nasonia vitripennis* is the best studied species, for which a wealth of information has been available on life-history and behavioural traits (e.g. Wylie 1966; Legner & Gerling 1967; Whiting 1967; Smith & Pimentel 1969; Davies 1975; Werren 1980, 1983a,b; King 1993; Rivers & Denlinger 1995; Drapeau & Werren 1999; Gadau *et al.* 1999; Bordenstein *et al.* 2001; Rivero & West 2002, 2005; Beukeboom & van den Assem 2004; Shuker & West 2004; Lalonde 2005; Shuker *et al.* 2005, 2007a,b; Bordenstein & Werren 2007; Pannebakker *et al.* 2008; Gadau & Wolschin 2009; Geuverink *et al.* 2009; Werren & Loehlin 2009; Raychoudhury *et al.* 2010a; Desjardins *et al.* 2010; Rivers *et al.* 2012; Niehuis *et al.* 2013; Gibson *et al.* 2013; Brucker & Bordenstein 2013). The release of its genome sequence led to the development of high-resolution genomic tools, for both interspecific (Niehuis *et al.* 2010; Loehlin *et al.* 2010a; Loehlin & Werren 2012; Desjardins *et al.* 2013) and intraspecific (Beukeboom *et al.* 2010; Pannebakker *et al.* 2010; Wang *et al.* 2013) research. The last years have seen a large number of studies that used the *Nasonia* genome sequence, reporting scientific breakthroughs in fundamental and applied biology (Loehlin *et al.* 2010b; Pannebakker *et al.* 2010, 2011; Verhulst *et al.* 2010; Werren *et al.* 2010; Lynch *et al.* 2011; Koevoets *et al.* 2012; Loehlin & Werren 2012; Brucker & Bordenstein 2013; Gibson *et al.* 2013; Niehuis *et al.* 2013; Paolucci *et al.* 2013). However, to further advance the utility of *N. vitripennis* as a genetic model system and to be able to fully exploit the advantages of its fully sequenced and annotated genome, a genetically variable and well-characterized experimental population, which is available for use in research worldwide, is highly required (e.g. Burke *et al.* 2010; Huang *et al.* 2012; Svenson *et al.* 2012).

In this study, we describe the establishment of the genetically diverse outbred HVRx laboratory population from strains that were collected from birds nest boxes and carrion baits in the Netherlands. Furthermore, we determine its (population) genetic characteristics and establish a breeding method that retains genetic variation over generations. To characterize the HVRx population, we describe the standing genetic variation and effective population size by microsatellite analysis. Pooled resequencing further yielded 417 331 high-quality SNPs, which are used for a genome-wide, high-resolution characterization of the population polymorphism. The HVRx population and its characterization are freely available as a community resource for investigators seeking to elucidate the genetic basis of complex trait variation using the *Nasonia* model system, for instance as a stock for association mapping in experimental evolution studies.

## Materials and methods

### HVRx population establishment and maintenance

In 2001, five *Nasonia vitripennis* strains were collected from different localities within 20 km radius from longitude 5.3 E and latitude 52.1 N in the Netherlands: Elspeet (strain ‘B5’), Bussum (strain ‘Bussum’) and the Hoge Veluwe (strains ‘HV55’, ‘HV287’ and ‘HV295’). Strains from the Hoge Veluwe were collected from bird nest boxes, while those from Elspeet and Bussum were collected from baits. Baits consisted of mesh screen bags, containing 25 laboratory hosts (*Calliphora vicina*) and a piece of liver. Each field strain was initiated by collecting all emerging female wasps from a nest box or bait and providing these with hosts. The number of founding females was not known, but host patches are usually colonized by multiple foundresses (Grillenberger *et al.* 2009b). After four generations of culturing (hosting about 100 females), ten mated females were randomly collected from each strain and individually provided with two hosts. From the offspring of each mated female, three virgin females and one male were isolated and mixed to initiate the HV population. Thus, a total 30 virgin females and 10 males from each strain were mixed in a 250-mL culture bottle and allowed to mate randomly. After 24 h, 200 hosts were added and the wasps were placed at 25 °C, under 16:8 light:dark conditions. After emergence, the HV population was split into two replicate populations (HV1 and HV2), by randomly isolating 100 mated females for each replicate and providing them with 200 hosts per replicate. Both replicates were maintained at 15 °C, 16:8 light:dark conditions.

**Fig 1:**
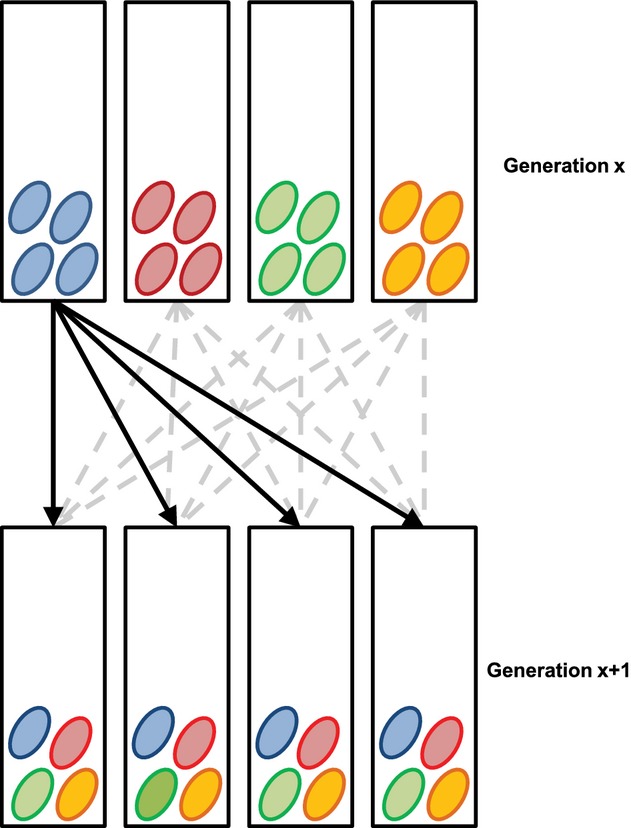
*Nasonia vitripennis * HVRx outbred laboratory population maintenance schedule. In each of four mass culture tubes, 40 mated female *N. vitripennis* wasps of generation *x* are provided with 50 *Calliphora* spp. hosts. After oviposition, the parasitized hosts are redistributed over four clean mass culture tubes, to ensure optimal mixing of the wasps over all four culture tubes and to allow mating between all wasps emerging within generation x + 1.

To maximize genetic diversity, we merged the HV1 and HV2 replicates to form one single HVRx population after approximately 36 generations of separate mass culturing. From both replicates, we hosted 80 mated females on 200 hosts, and the offspring was allowed to mate randomly before dividing the wasps over four mass culture tubes (70 × 20 mm) each containing 50 fly hosts for oviposition. After 1 week, the parasitized hosts were distributed over four new mass culture tubes. After emergence, approximately 30 mated females from each mass culture tube were transferred to new mass culture tubes to initiate the next generation (Fig. 1). This breeding procedure ensures the maintenance of a large outbred population by allowing random mating between wasps of different tubes and was designed to preserve genetic diversity across generations. The HVRx population was maintained on *C. vicina* pupae as hosts, at 25 °C, 16: 8 light:dark conditions.

### Microsatellite analysis

To determine and monitor the genetic diversity of the HVRx population over time, we isolated DNA from females from the HV1 (*n* = 14) and HV2 (*n* = 14) strains, and from the HVRx population in generations 1 (*n* = 12), 5 (*n* = 24), 10 (*n* = 24) and 32 (*n* = 24), using a standard high-salt-chloroform protocol (Maniatis *et al.* 1982). A set of 40 microsatellite markers, evenly distributed over the five chromosomes of *N. vitripennis* and combined into six multiplex sets, was used to determine genetic variation (Beukeboom *et al.* 2010; Pannebakker *et al.* 2010; Koevoets *et al.* 2012). Details of these microsatellite markers are listed in Table S1 (Supporting information). Microsatellite markers were amplified using the Qiagen multiplex PCR kit (Qiagen, Hilden, Germany) according to the manufacturer's recommendations (PCR profile: 15 min at 95 °C, followed by 30 cycles of 30 s at 94 °C, 1.5 min at annealing temperature and 1 min at 72 °C, followed by 45 min at 72 °C). Amplification was in 5 *μ*L volumes using Applied Biosystems Veriti or Applied Biosystems 9700 thermocyclers (Applied Biosystems, Foster City, CA, USA). Fragments were diluted 400 times, separated on a Applied Biosystems 3730 DNA Analyzer and analysed using GeneMapper v4.0 (Applied Biosystems). Population genetic statistics (allelic richness *R*, heterozygosity *H*_E_, *F*_ST_) were calculated using fstat 2.9.3 (Goudet 2001). Statistical analyses were carried out using r 2.14.1 (R Development Core Team 2012).

### Theoretical estimate of effective population size

To evaluate the expected effective population size (*N*_e_) achieved by our set-up, we simulated a population propagated under the four-tube breeding schedule that was used for maintenance of the HVRx population, as well as a population of identical census size but propagated as a single large one (i.e. in a single large tube). We modelled the sampling of a single biallelic locus in a haplodiploid system with 30 (four-replicates) or 120 (single-replicate) mated females per tube, to match the approximate number of mated females transferred each generation. For each replicate, mating between diploid females and haploid males was simulated, with females producing offspring from a single male selected with replacement from a vector of males of length *rN*_f_, where *N*_f_ is the number of females and *r* is the observed male over female ratio. For the multiple-replicate scheme, mated females from different tubes were mixed in equal proportions into new tubes (following Fig. 1).

For each simulation, we calculated *F*_*t*_, the allelic differentiation with respect to the founding population, as: 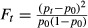 (Nicholson *et al.* 2002), where *p*_*t*_ is the allele frequency in generation *t* and *p*_*0*_ is the frequency in the founding population. *N*_e_ was then estimated by solving the following nonlinear equation:
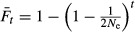
, where 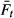 was obtained by averaging *F*_*t*_ over 100 000 independent simulations. This simulated estimate of *N*_e_ was compared with the classic theoretical prediction of inbreeding effective size in a haplodiploid populations derived by Wright (1933) as


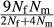


where *N*_f_ and *N*_m_ are the number of females and the number of males, respectively.

### Empirical estimation of effective population size

We used approximate Bayesian computation (ABC, Tavare *et al.* 1997; Marjoram *et al.* 2003) to obtain estimates of the posterior distribution of realized *N*_e_ over the past 32 generations from the microsatellite data. We estimated the following summary statistics from the data in generation 5, 10 and 32: number of alleles, expected heterozygosity and *F*_*t*_ (as defined above and averaged over all loci and alleles). This resulted in a vector of length 9, *O*, with each element *O*_*ij*_ containing the observed value for summary statistic *i* in generation *j*. We then simulated 10 000 000 instances of multilocus genotypes sampled from a diploid Wright–Fisher population subject to drift over 32 generations, with proposal values of *N*_e_ drawn from a uniform distribution between 0 and 1000. Starting allele frequencies were set to match those observed in the base population. In each independent simulation, the same summary statistics as above were measured in generation 5, 10 and 32, yielding a vector *S* of simulated summary statistics, with each element *S*_*ij*_ corresponding to a value in *O*_*ij*_. Following Pritchard *et al*. (1999), we only accepted iterations for which | *S*_*ij*_ _–_ *O*_*ij*_ |/*O*_*ij*_ < *ε* for each element of *S*_*ij*_ and *O*_*ij*_. The threshold value *ε* was set to 0.11 to achieve an acceptance rate of 0.001.

### Genome resequencing and analysis

To resequence the HVRx base population, DNA from 20 pooled females from generation four was extracted and purified using Qiagen DNeasy columns (Qiagen). A 101-bp unpaired and a 76-bp paired-end library were constructed and subsequently sequenced by the University Medical Centre Groningen (UMCG) Genome Analysis Facility according to standard Illumina protocols. The unpaired library was run on a single lane and the paired-end library on five lanes of an Illumina Genome Analyzer II (Illumina, San Diego, CA, USA). Sequence data were provided to NasoniaBase (Munoz-Torres *et al.* 2011) and submitted to the NCBI Short Read Archive with accession no. SRP022050.

Reads were aligned to the v2.0 of the *N. vitripennis* genome (Werren *et al.* 2010) using Mosaik Aligner v1.1.0021 (https://code.google.com/p/mosaik-aligner/). SNPs were filtered from the HVRx assembly using Varscan.v2.2.8 (Koboldt *et al.* 2012), according to the following rules: minimum consensus quality: 20; minimum count of minor allele: 2, minimum coverage: 8. Reads with mapping qualities less than 20 were discarded. SNP data were provided to NasoniaBase (Munoz-Torres *et al.* 2011) and deposited in Dryad (Dryad Entry doi:10.5061/dryad.f2330).

Mean SNP densities were calculated in nonoverlapping 100 kb windows, with a minimum mean coverage of 8. Genome-wide diversity analysis was performed using the popoolation pipeline (Kofler *et al.* 2011). We estimated nucleotide diversity *π* in nonoverlapping 100 kb windows, using the same parameters augmented with a maximum coverage of 1 000 000, a fraction of the window that has a coverage between the minimum and maximum coverage of 0.6. Additional statistical analyses were carried out using r 2.14.1 (R Development Core Team 2012).

## Results

After being maintained in the laboratory for approximately 36 generations, the HV1 and HV2 populations had genetically diverged (*F*_ST_ = 0.26, *P* < 0.05, Table1). The mean heterozygosity was not statistically different between both replicates (mean *H*_E_* *± SE HV1 = 0.53 ±0.03, HV2 = 0.46 ± 0.05, *t*_74_* *=* *1.41, *P* = 0.163, Table2), which is reflected in the heterozygosity per locus for HV1 and HV2 (Table S2, Supporting information). The mean allelic richness did show differences between both replicate populations (mean *R *± SE HV1 = 3.08 ± 0.18, HV2 = 2.52 ± 0.14, *t*_78_* *=* *2.50, *P* = 0.015, Table2).

**Table 1:** Genetic differentiation in the *Nasonia vitripennis* HVRx outbred laboratory population. Pairwise *F*_ST_ values between HV1 and HV2 founder populations and generations 1, 5, 10 and 32 of the HVRx population over 40 microsatellite loci combined

	HV1	HV2	HVRx-G1	HVRx-G5	HVRx-G10	HVRx-G32
HV1	0.00	0.26*	0.04*	0.04*	0.06*	0.11*
HV2		0.00	0.16*	0.17*	0.18*	0.22*
HVRx-G1			0.00	0.02	0.03*	0.07*
HVRx-G5				0.00	0.03*	0.06*
HVRx-G10					0.00	0.05*
HVRx-G32						0.00

* Significant values *P* < 0.05 after Bonferroni correction following G-statistics (as implemented in Fstat).

**Table 2:** Genetic variation in the *Nasonia vitripennis* HVRx outbred laboratory population

	Expected heterozygosity *H*_E_	Allelic richness *R*
HV1	0.53 (0.03)^a^	3.08 (0.18)^ab^
HV2	0.46 (0.04)^ab^	2.52 (0.14)^a^
HVRx-G1	0.58 (0.04)^a^	3.52 (0.22)^b^
HVRx-G5	0.60 (0.03)^ac^	3.45 (0.20)^b^
HVRx-G10	0.56 (0.03)^a^	3.40 (0.18)^b^
HVRx-G32	0.56 (0.03)^a^	3.34 (0.21)^b^

Table shows the mean expected heterozygosity *H*_E_ and the mean allelic richness *R*. Standard errors are in parentheses.

Different lowercase letters indicate significant differences at *P* < 0.05 for each estimate.

The HV1 and HV2 replicates were merged to start the HVRx population with maximum genetic diversity. We subsequently cultured the HVRx population following a breeding procedure designed to retain genetic diversity over generations (Fig. 1). To confirm the effectiveness of our breeding procedure, we monitored the population genetic characteristics of the HVRx population at generations 1, 5, 10 and 32 after establishment.

### Genetic differentiation

Already at generation 1, the HVRx population had significantly diverged from both original populations (*F*_ST_ = 0.04 (HV1) and 0.16 (HV2), *P* < 0.05, Table1). After generation 5, the HVRx population started to diverge modestly but significantly between subsequent generations (Table1). However, 32 generations of controlled breeding resulted in a *F*_ST_ value of 0.07 between generation 1 and generation 32, which is considerably lower than that of the differentiation between the original HV1 and HV2 populations after 36 generations (Table1).

### Genetic variation

Mean heterozygosities (*H*_E_ ± SE) for HVRx generations 1, 5, 10 and 32 did not change and were 0.58 ± 0.04, 0.60 ± 0.03, 0.56 ± 0.03 and 0.56 ± 0.03, respectively. An overall comparison, including also the founding populations HV1 and HV2, showed that the only significant difference in heterozygosity was between HV2 and HVRx generation 5 (Tukey's HSD critical value = 0.127, Table2). Our results indicate that heterozygosity was effectively maintained over 32 generations. This pattern is also reflected in the per-locus heterozygosity (Table S2, Supporting information).

Mean allelic richness (*R* ± SE) showed a slight decrease over generations 1, 5, 10 and 32 and was 3.52 ± 0.22, 3.45 ± 0.20, 3.40 ± 0.18 and 3.34 ± 0.21, respectively. An overall comparison, including also the founding populations HV1 and HV2 only, showed significant differences in allelic richness between HV2 and all HVRx generations (One-way aov; *F*_5,234_ = 3.87, *P* = 2.170e-3; Tukey HSD critical value = 0.741, Table2). Overall, our results indicate that our controlled breeding method effectively limits the decrease in allelic richness over 32 generations.

### Effective population size

**Fig 2:**
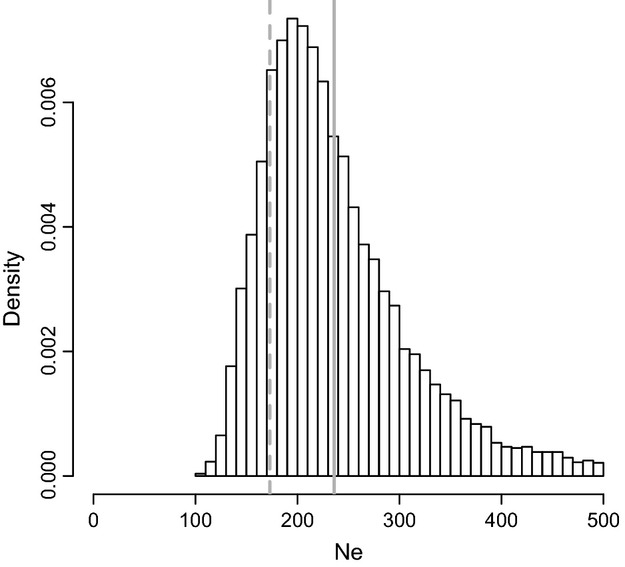
Effective population size *N*_e_ in the *Nasonia vitripennis* HVRx outbred laboratory population. Posterior density of the empirical estimate of *N*_e_ based on 40 microsatellite loci, obtained by approximate Bayesian computation. Blue, red and green lines indicate the theoretical prediction based on Wright (1933), the posterior mean and posterior median of the empirical estimate, respectively.

The empirical estimation of effective population size (*N*_e_), based on the diversity parameters above, yielded a posterior mode of 195 and a mean of 236 diploid individuals. The computer simulations of the different breeding schemes yielded nearly identical effective population sizes of *N*_e_ = 177 and *N*_e_ = 173 for the four-replicate and single-replicate mating scheme, respectively, entirely consistent with the theoretical prediction of *N*_e_ = 173 and falling within 0.9 and 0.3 standard deviations of the posterior mean and mode, respectively (Fig. 2).

### Genomic variation

**Fig 3:**
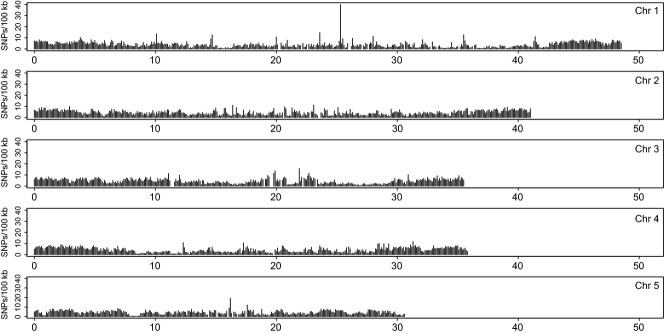
Genome-wide SNP density pattern in the *Nasonia vitripennis* HVRx outbred laboratory population. Mean SNP density adjusted for read depth in nonoverlapping 100 kb windows, plotted against chromosomal position.

We found considerable genetic variability in the HVRx population, with a total of 417 331 SNPs distributed over the five chromosomes, or about 1 SNP per 796.7 bp [based on a physical genome size of 332.5 Mb (Gregory, 2013)]. SNP density varied between the chromosomes (one-way aov; *F*_4,1880_ = 9.58, *P* = 1.16e-07, Table3), with the lowest mean density per 100 kb window on chromosome 1. The pattern in SNP density differentiation is retained (one-way aov; *F*_4,1880_ = 1105.1, *P* < 2.2e-16, Table3, Fig. 3) upon adjusting for differences in read depth between the different chromosomes (one-way aov; *F*_4,1880_ = 12.268, *P* = 7.55e-10, Table3, Fig. S1, Supporting information). Interestingly, the highest SNP density per 100 kb window was also found on chromosome 1 at 25.3 Mb showing 438 SNPs (or 39.84 SNPS after adjusting for 10.99X read depth).

**Table 3:** Genomic variation per chromosome in the *Nasonia vitripennis* HVRx outbred laboratory population. Mean SNP density, read depth adjusted SNP density, read depth and nucleotide diversity per chromosome in 100 kb nonoverlapping sliding windows

	Mean SNP density in 100 kb windows (SE)	Mean read depth adjusted SNP density in 100 kb windows (SE)	Mean read depth per 100 kb window (SE)	Mean nucleotide diversity (*π*) in 100 kb windows (SE)
1	179.57 (5.83)^a^	3.96 (0.13)^a^	45.05 (0.71)^a^	0.0011 (4.00e−05)^a^
2	219.10 (5.74)^bc^	4.43 (0.10)^bc^	49.32 (0.64)^b^	0.0014 (3.54e−05)^b^
3	214.99 (6.44)_bd_	4.53 (0.13)^bd^	47.95 (0.79)^bc^	0.0013 (4.30e−05)^b^
4	197.96 (6.62)_acd_	4.21 (0.12)^acd^	46.90 (0.70)^ac^	0.0013 (4.54e−05)^b^
5	224.59 (6.34)^b^	4.37 (0.12)^b^	51.07 (0.70)^b^	0.0014 (3.85e−05)^b^
All	202.29 (2.80)	4.28 (0.06)	47.84 (0.32)	0.0013 (1.83e−05)

Standard errors are in parentheses.

Different lowercase letters indicate significant differences at *P* < 0.05 for each estimate.

**Fig 4:**
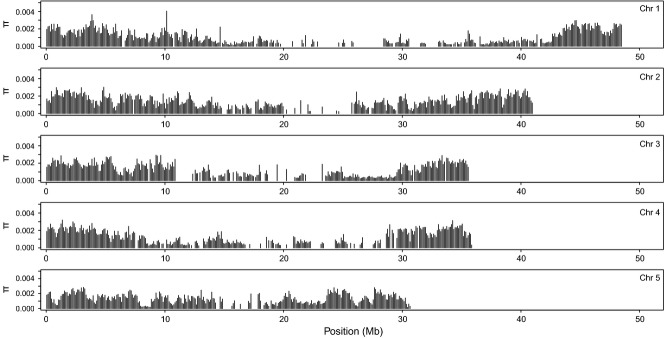
Genome-wide polymorphism pattern in the *Nasonia vitripennis* HVRx outbred laboratory population. Mean estimates of nucleotide diversity (*π*) in nonoverlapping 100 kb windows, plotted against chromosomal position.

The mean nucleotide diversity per 100 kb window over all chromosomes was 0.13%, showing significant differentiation by chromosome (one-way aov; *F*_4,155_ = 9.97, *P* = 5.76e-08, Table3), with chromosome 1 showing the lowest nucleotide diversity compared with other chromosomes. The distribution of genomic variation over the chromosomes showed similar patterns for both SNP density as for nucleotide diversity. Both measures were higher towards the tips of the chromosomes than the centres (Figs3 and 4). Estimates of nucleotide diversity were lacking in areas where the read depths were low, such as the near the centre of the chromosomes (Fig. 4).

## Discussion

The HVRx laboratory population captures a large amount of genetic variation. With over 400 000 SNPs, it is a valuable resource for investigating the genetic architecture of complex trait variation in the *Nasonia* genetic model system. The HVRx population provides a genetically variable stock population for experimental evolution experiments, in which the availability of genomic information enables efficient association mapping of complex traits. While genetically diverse stock populations are available for other model species (*Drosophila*: Burke *et al.* 2010; Huang *et al.* 2012; mouse: Svenson *et al.* 2012; *Arabidopsis*: Kover *et al.* 2009), HVRx is the first available for the Hymenoptera genetic model *Nasonia*.

The HVRx population is maintained at a breeding schedule that retains the available genetic variation, evidenced by the fact that over 32 generations, both the heterozygosity *H*_E_ and the allelic richness *R* had not changed significantly. While there was greater differentiation between both founding HV replicates (*F*_ST_ = 0.26) than between European and North American *Nasonia vitripennis* populations (*F*_ST_ = 0.15, Raychoudhury *et al.* 2010a), our breeding schedule effectively prevented further differentiation over generations (Table1). Using a simple breeding procedure, our set-up results in a simulated population size of *N* = 177, and an effective population size *N*_e_ of 236. This equals to an *N*_e_/*N* ratio close to 1.5, which is high compared with previously reported estimates for natural and captive populations of other species (Briscoe *et al.* 1992; Frankham 1995; Simoes *et al.* 2010). While effective population size is influenced by many factors, such as fluctuations in population size over generations, variation in family size and sex ratio, mating system and selection, work by Simoes *et al*. (2010) has shown that careful maintenance procedures can efficiently reduce the loss of genetic variability in laboratory populations in organisms that generate a high number of offspring. The results described here for the HVRx population corroborate this finding.

The effective population size is an important parameter for predicting the potential response to selection (Falconer & Mackay 1996). Because the effect of drift is reduced in larger populations, allelic diversity persists longer allowing for greater and faster responses to selection (Weber 2004). While the exact response to selection is determined by intensity of selection, heritability of the trait of interest and effective population size, the empirical estimate of *N*_e_ = 236 for HVRx is on upper range used in artificial selection studies (Weber & Diggins 1990; Weber 2004; Zhang & Hill 2005). The population sizes of laboratory populations also received attention in the biological control literature, where the maintenance of genetic variability is an important issue. The population size of the HVRx exceeds the general recommendation to start and maintain natural enemy cultures with an effective population size of *N*_e_* *> 100 (Roush 1990; Bartlett 1993; Nunney 2003).

The genetic diversity of the HVRx laboratory population is slightly lower than that observed in studies of natural *N. vitripennis* populations. Evaluating 12 overlapping microsatellite loci, the heterozygosity of the HVRX population after 32 generations (mean 0.52 ± 0.07) is lower than that observed in natural *N. vitripennis* populations directly upon collection from the field (range of *H*_E_: 0.46–0.95 Table S3, Supporting information; Grillenberger *et al.* 2009a; Raychoudhury *et al.* 2010b; Paolucci *et al.* 2013). It is, however, considerably higher than in the highly inbred standard laboratory strain AsymCX (Werren *et al.* 2010). A similar pattern is observed in the genome-wide nucleotide diversity of the HVRx population (*π* = 0.0013), which is lower than the levels of synonymous nucleotide diversity (ranging from *π* = 0.0016 to 0.0026) observed in ∼16 kb sequence from 27 genes in multiple strains of *N. vitripennis* (Raychoudhury *et al.* 2010b; Werren *et al.* 2010). The genome-wide nucleotide diversity of the HVRx population, however, included both synonymous and nonsynonymous polymorphisms, which result in a lower estimate than when just synonymous polymorphisms were used. Overall, the levels of nucleotide diversity in *Nasonia* are much lower than those observed in natural *Drosophila melanogaster* populations (e.g. Mackay *et al.* 2012; Orozco-ter Wengel *et al.* 2012), which could be explained by founder events, but also by effects more specific to haplodiploids such as the purging of deleterious mutations in haploid males or high levels of inbreeding (Hedrick & Parker 1997).

Analysis of the genomic variation in the HVRx population shows that genomic polymorphism, both in terms of SNP density as well as of nucleotide diversity, is lower in the centre of the chromosomes and higher towards the distal ends. While we have no exact data on the location of the centromeres, the fact that all *Nasonia* chromosomes are meta- or submetracentric (Gokhman & Westendorff 2000) strongly suggests the centromeres are at or near the centre of the chromosomes. These regions not only show reduced nucleotide diversity in the HVRx population, but also show reduced recombination rates in *Nasonia* (Niehuis *et al.* 2010; Desjardins *et al.* 2013), consistent with the correlation between recombination rate and nucleotide diversity observed in a wide range of organisms (Begun & Aquadro 1992; Nachman 2002). Unfortunately, the nucleotide diversity data are not complete throughout the HVRx genome, due to low read depths at several regions. While we cannot exclude sequence quality issues for these regions, low read depth regions colocate with gaps in the Nvit 2.0 reference sequence (Werren *et al.* 2010) that make reference mapping impossible in those regions.

The HVRx laboratory population is a genetically diverse *N*. *vitripennis* outbred laboratory population that fills a gap between conventional inbred *Nasonia* laboratory strains and free-living natural populations. It offers higher levels genetic variation than inbred laboratory strains and easier experimental manipulation than free-living populations, by capturing intraspecific genetic variation in a tractable laboratory population that is easily maintained using the provided breeding schedule. While inbred laboratory strains are a valuable tool for studying interspecific differences in *Nasonia* (e.g. Desjardins *et al.* 2010; Werren *et al.* 2010; Koevoets *et al.* 2012; Loehlin & Werren 2012; Gibson *et al.* 2013; Niehuis *et al.* 2013), the intraspecific context is crucial to understand the evolutionary forces underlying adaptation of complex (life-history) traits. The HVRx adds a novel resource for both inter- and intraspecific research in *Nasonia*, thereby further extending the qualities of the *Nasonia* genetic model system for developmental and evolutionary research. The combination of a genetically diverse outbred population with full genomic information makes HVRx a solid starting point for studying the genomic changes underlying the evolution of complex traits, for instance as a stock population for experimental evolution in combination with association mapping studies.

## Data Accessibility

Sequence data were provided to NasoniaBase (http://hymenopteragenome.org/Nasonia/) and submitted to the NCBI Short Read Archive with accession no. SRP022050. SNP data were provided to NasoniaBase and deposited in Dryad (Dryad Entry doi:10.5061/dryad.f2330).

## Supporting Information

Fig S1 Read depth in the *N. vitripennis* HVRx outbred laboratory population. **Table S1** Microsatellite marker properties. **Table S2** Expected heterozygosities per microsatellite marker. **Table S3** Heterozygosity in HVRx generation 32 and natural *N. vitripennis* populations.
