# Supplementary material for: Development of a Nasonia vitripennis outbred laboratory population for genetic analysis
Source: Mol Ecol Resour. 2013 Dec 12;14(3):578–87. doi: 10.1111/1755-0998.12201 (PMC4260118; doi:10.1111/1755-0998.12201)
Supplement: Fig S1 — Read depth in the N. vitripennis HVRx outbred laboratory population. Table S1 Microsatellite marker properties. Table S2 Expected heterozygosities per microsatellite marker. Table S3 Heterozygosity in HVRx generation 32 and natural N. vitripennis populations. [file men0014-0578-SD1.docx]

**Development of a *Nasonia vitripennis* outbred laboratory population for genetic analysis**

Louis van de Zande^1^, Steven Ferber^1^, Ammerins de Haan^1^, Leo W. Beukeboom^1^, Joost van Heerwaarden^2^, Bart A. Pannebakker^1,3^

^1^Evolutionary Genetics, Centre for Ecological and Evolutionary Studies, University of Groningen, 9700 CC, Groningen, The Netherlands

^2^Biometris, Wageningen UR, 6708 PB, Wageningen, The Netherlands

^3^Laboratory of Genetics, 6708 PB, Wageningen University, Wageningen, The Netherlands

Corresponding author: B.A. Pannebakker, Wageningen University, Laboratory of Genetics, Droevendaalsesteeg 1, 6708 PB Wageningen, The Netherlands. Email: bart.pannebakker@wur.nl

**Figure S1 Read depth in the *N. vitripennis* HVRx outbred laboratory population.** Mean read depth per 100kb sliding window over each chromosome.

**Table S1 Microsatellite marker properties.** Table shows marker name, chromosome and position, primer sequence, 5’ fluorescent label, multiplex pool and source of the sequence.

| Marker | Chromosome | Position (cM) | Primer sequence (5' to 3') | 5' Label | Multiplex pool | Source^1^ |
| --- | --- | --- | --- | --- | --- | --- |
| Nv200 | 1 | 0 | TCCGCACGGTCAGTCTTT | 6FAM | 1 | FJ156190.1 |
|  |  |  | CGGCGAATTTCGTTCTTC |  |  |  |
| Nv308 | 1 | 16.3 | ATTCGGAATCCACGAAACG | HEX | 4 | FJ555533.1 |
|  |  |  | TAGGGCGCGTATAGATCGAG |  |  |  |
| Nv202 | 1 | 29.9 | GGCTTCTCGCCATAATGAG | NED | 4 | FJ156192.1 |
|  |  |  | GCCGTATTGGCTCTCGTCTA |  |  |  |
| Nv121 | 1 | 37.1 | AATTCATAAGCCAAGCTCACG | NED | 6 | FJ156122.1 |
|  |  |  | TCATCGCTCTGGCAATTTTT |  |  |  |
| Nv311 | 1 | 50.3 | ACTGGCGAAAGCTCAAACC | 6FAM | 2 | FJ156219.1 |
|  |  |  | TCGAGCTTTGTTCTGGGATA |  |  |  |
| Nv106 | 1 | 80.5 | GCAACGAGATTAACCCTTG | HEX | 2 | FJ156230.1 |
|  |  |  | GGCTTTCTAGATGGCGACTC |  |  |  |
| Nv205 | 1 | 97.8 | GCTCGAGGAGGCCATATC | 6FAM | 1 | FJ156195.1 |
|  |  |  | CCTCGATAGCTGGCAACC |  |  |  |
| Nv211 | 2 | 0 | TGAAGAATGCGTATCAATCGTAC | HEX | 5 | FJ156201.1 |
|  |  |  | ACCACCACGTCCTCCAAG |  |  |  |
| Nv305 | 2 | 14 | ATTTACGCTCCTGCGAGACA | HEX | 6 | FJ156215.1 |
|  |  |  | CCGCTTCGAAAGGAGATATG |  |  |  |
| Nv210 | 2 | 20.4 | AGGACGCAGCTAGGTGGC | 6FAM | 2 | FJ156200.1 |
|  |  |  | CCTCGTCGATCAAGAGGC |  |  |  |
| Nv301 | 2 | 33 | CGAGGCAACGATTTTCTTTC | 6FAM | 2 | FJ156212.1 |
|  |  |  | CGTATCGCACTGCTTGTGTT |  |  |  |
| Nv209 | 2 | 39.8 | CCAACTTCTTATTCGTAAGGGAA | HEX | 1 | FJ156199.1 |
|  |  |  | ACCATTCGCTGGCTGGTA |  |  |  |
| Nv306 | 2 | 48.1 | TGCTCGGATTTCGAACATTT | 6FAM | 1 | FJ156216.1 |
|  |  |  | GCGGATGTTGTTCCGTTATT |  |  |  |
| Nv208 | 2 | 66.6 | GCACCGCTGCGATTAAAC | HEX | 1 | FJ156198.1 |
|  |  |  | TGCTCTCGCTTCTCGAGTC |  |  |  |
| Nv300 | 2 | 79.5 | ACATTCCGCAGAGCGATTAT | 6FAM | 3 | FJ156211.1 |
|  |  |  | CGCGACCGATGATTTACTC |  |  |  |
| Nv207 | 2 | 88.4 | GCAGACACTGACACGTGATG | 6FAM | 4 | FJ156197.1 |
|  |  |  | CCCTCGATCGCTGCACTA |  |  |  |
| Nv206 | 2 | 103.7 | CGATGTTGCGACCGTCTATA | HEX | 5 | FJ156196.1 |
|  |  |  | TCCGATCAAATCGAATTACTGTA |  |  |  |
| Nv212 | 3 | 0 | CATAAATACATTTGGGTCTCCC | NED | 2 | FJ156202.1 |
|  |  |  | TGGAGTCCAGCTAGGATTCTAA |  |  |  |
| Nv319 | 3 | 12.6 | TTTGAGGTTATGCGTCGTTTC | HEX | 3 | FJ156222.1 |
|  |  |  | GAGCGGAGTGCTTCATTCAG |  |  |  |
| Nv213 | 3 | 21.9 | GGAAGGAGCGAATCCTCTAC | HEX | 3 | FJ156203.1 |
|  |  |  | AGTGCGTCTCGACGCTAG |  |  |  |
| Nv303 | 3 | 39.8 | GACAATAGCCGCTACGGAAA | HEX | 3 | FJ156214.1 |
|  |  |  | CGTCGTTCTGCTGCTTGTC |  |  |  |
| Nv312 | 3 | 47.6 | GCACACACTCGCGATAAGAA | HEX | 6 | FJ156220.1 |
|  |  |  | TGTAGAATTCGCCGTGTGAC |  |  |  |
| Nv215 | 3 | 60.1 | CACGAAACTACATCGCAATCA | 6FAM | 2 | J. Gadau, pers. comm. |
|  |  |  | CGTGTATAGCTGCTCTTGTTGAA |  |  |  |
| Nv216 | 3 | 74.9 | CGCTCTCTGCGTGTGTCT | HEX | 4 | J. Gadau, pers. comm. |
|  |  |  | AGAGCGATAGCGTCGCTT |  |  |  |
| Nv217 | 3 | 97.4 | AATGGCATTATGCGAATGA | 6FAM | 5 | J. Gadau, pers. comm. |
|  |  |  | CTGCTCTCTGCATGAATCTTT |  |  |  |
| Nv218 | 4 | 0 | TCGCTTAGATAATTGCCAGAC | 6FAM | 3 | J. Gadau, pers. comm. |
|  |  |  | ACAGATATACTCTCGTGCAGGAG |  |  |  |
| Nv112 | 4 | 16.5 | GCTCCTCCTTGTTTGCGTTA | 6FAM | 1 | FJ156116.1 |
|  |  |  | TCGAGCGACGAGTGATATCTT |  |  |  |
| Nv219 | 4 | 22.4 | GCCTGCCGTACAATCAAA | NED | 1 | J. Gadau, pers. comm. |
|  |  |  | GAAACGCGACGCTGTTAG |  |  |  |
| Nv118 | 4 | 37.5 | AGAATCGAAGCGGGATTAGC | HEX | 6 | FJ156232.1 |
|  |  |  | TTAAATCCCAGCCAGACGAG |  |  |  |
| Nv114 | 4 | 48.6 | ATGGGCAATAAAACGAAACG | HEX | 4 | FJ156231.1 |
|  |  |  | CATCCTTGCGGAGACACTAA |  |  |  |
| Nv309 | 4 | 56.9 | TTCAGCTTCACGCTCAGGTA | 6FAM | 4 | FJ156218.1 |
|  |  |  | GCGAGAGCAATCAGAAACAA |  |  |  |
| Nv221 | 4 | 64.5 | GTCCGTGTGTACTGCGAAG | HEX | 6 | J. Gadau, pers. comm. |
|  |  |  | ACCTCGGAAACGGCTAGA |  |  |  |
| Nv321 | 4 | 89.3 | CGGTGAGACTCGTGAGATGA | HEX | 5 | FJ156224.1 |
|  |  |  | AACCGCAGCTCTCAACATTT |  |  |  |
| Nv223 | 4 | 103.1 | CGGATATCTACGGAAATAGCATT | 6FAM | 6 | J. Gadau, pers. comm. |
|  |  |  | AACACACTCGCTCGCTTT |  |  |  |
| Nv224 | 5 | 0 | CGGCATACCTATAGCGCAGA | NED | 5 | J. Gadau, pers. comm. |
|  |  |  | CCATCCATTCGGAATACAATCT |  |  |  |
| Nv125 | 5 | 26.5 | TGGCATCACGCTCTAAAATG | 6FAM | 5 | FJ156126.1 |
|  |  |  | GCGCGTGCATCTATATGTGT |  |  |  |
| Nv109 | 5 | 42.7 | GCTTACTCTCGGGAACTGGA | 6FAM | 3 | FJ156114.1 |
|  |  |  | CGAGCATTAACCATCAGCAG |  |  |  |
| Nv322 | 5 | 55 | CGAAAGAAGCCAAGCATAGAA | 6FAM | 5 | FJ156225.1 |
|  |  |  | GAGAAAAATCGGGTCGAAGT |  |  |  |
| Nv228 | 5 | 73.6 | ACGCATAAAAGTTGCACACG | 6FAM | 4 | J. Gadau, pers. comm. |
|  |  |  | GGTGGATGGCTTCTCGTATT |  |  |  |
| Nv229 | 5 | 94.4 | AAATATTGGCGCGGCAAC | NED | 3 | J. Gadau, pers. comm. |
|  |  |  | CCAACAATGAGTGTATCCTAGGC |  |  |  |

^1^ Genbank Accesion number or primer source

**Table S2 Expected heterozygosities per microsatellite marker.** for each of 38 microsatellite markers. Table shows marker name, chromosome and position in centiMorgan, expected heterozygosity (*H_E_*; gene diversity) for HV1 and HV2, and HVRx in generations 1,5,10 and 32 after merging HV1 and HV2. For each sample point, the mean expected heterozygosity (± standard error) is given.

| Marker | Chromosome | Position (cM) | HV1 | HV2 | HVRx-G1 | HVRx-G5 | HVRx-G10 | HVRx-G32 |
| --- | --- | --- | --- | --- | --- | --- | --- | --- |
| Nv200 | 1 | 0 | 0.731 | 0.574 | 0.53 | 0.71 | 0.752 | 0.689 |
| Nv308 | 1 | 16.3 | 0.451 | 0.302 | 0.159 | 0.223 | 0.12 | 0.083 |
| Nv202 | 1 | 29.9 | 0.606 | 0.679 | 0.595 | 0.654 | 0.466 | 0.379 |
| Nv121 | 1 | 37.1 | 0.577 | 0.625 | 0.446 | 0.672 | 0.755 | 0.795 |
| Nv311 | 1 | 50.3 | 0.371 | 0.797 | 0.553 | 0.575 | 0.448 | 0.552 |
| Nv106 | 1 | 80.5 | 0.737 | 0.676 | 0.6 | 0.887 | 0.771 | 0.907 |
| Nv205 | 1 | 97.8 | 0.641 | 0.538 | 0.765 | 0.593 | 0.775 | 0.719 |
| Nv211 | 2 | 0 | 0.318 | 0.449 | 0.361 | 0.755 | 0.483 | 0.675 |
| Nv305 | 2 | 14 | 0 | 0.308 | 0.235 | 0.24 | 0.211 | 0.192 |
| Nv210 | 2 | 20.4 | 0.78 | 0.552 | 0.826 | 0.745 | 0.694 | 0.448 |
| Nv301 | 2 | 33 | 0.544 | 0.489 | 0.508 | 0.505 | 0.442 | 0.284 |
| Nv209 | 2 | 39.8 | 0.676 | 0.621 | 0.773 | 0.739 | 0.753 | 0.736 |
| Nv306 | 2 | 48.1 | 0.494 | 0.302 | 0.508 | 0.467 | 0.504 | 0.491 |
| Nv208 | 2 | 66.6 | 0.077 | 0.516 | 0.47 | 0.408 | 0.357 | 0.623 |
| Nv300 | 2 | 79.5 | 0.253 | 0 | 0 | 0.19 | 0.05 | 0.156 |
| Nv207 | 2 | 88.4 | 0.67 | 0.646 | 0.784 | 0.699 | 0.646 | 0.62 |
| Nv206 | 2 | 103.7 | 0.673 | 0.69 | 0.792 | 0.673 | 0.764 | 0.746 |
| Nv212 | 3 | 0 | 0.522 | 0.415 | 0.545 | 0.48 | 0.546 | 0.204 |
| Nv319 | 3 | 12.6 | 0.758 | 0.374 | 0.856 | 0.823 | 0.607 | 0.654 |
| Nv213 | 3 | 21.9 | 0.071 | 0.511 | 0.159 | 0.294 | 0.23 | 0.449 |
| Nv303 | 3 | 39.8 | 0.429 | 0 | 0.439 | 0.475 | 0.359 | 0.495 |
| Nv215 | 3 | 60.1 | 0.39 | 0 | 0.227 | 0.43 | 0.198 | 0.409 |
| Nv216 | 3 | 74.9 | 0.736 | 0.522 | 0.705 | 0.755 | 0.735 | 0.735 |
| Nv217 | 3 | 97.4 | 0.519 | 0.684 | 0.386 | 0.688 | 0.53 | 0.636 |
| Nv218 | 4 | 0 | 0.75 | 0.657 | 0.791 | 0.769 | 0.723 | 0.571 |
| Nv112 | 4 | 16.5 | 0.455 | 0.137 | 0.636 | 0.516 | 0.559 | 0.663 |
| Nv219 | 4 | 22.4 | 0.676 | 0.602 | 0.777 | 0.751 | 0.843 | 0.691 |
| Nv118 | 4 | 37.5 | 0.635 | 0.665 | 0.822 | 0.687 | 0.744 | 0.658 |
| Nv114 | 4 | 48.6 | 0.514 | 0.071 | 0.663 | 0.582 | 0.613 | 0.694 |
| Nv221 | 4 | 64.5 | 0.434 | 0.456 | 0.686 | 0.592 | 0.641 | 0.557 |
| Nv321 | 4 | 89.3 | 0.426 | 0.577 | 0.64 | 0.584 | 0.649 | 0.749 |
| Nv223 | 4 | 103.1 | 0.61 | 0.365 | 0.814 | 0.74 | 0.709 | 0.563 |
| Nv224 | 5 | 0 | 0.541 | 0.604 | 0.803 | 0.73 | 0.719 | 0.511 |
| Nv125 | 5 | 26.5 | 0.69 | 0 | 0.659 | 0.62 | 0.526 | 0.663 |
| Nv109 | 5 | 42.7 | 0.684 | 0.538 | 0.788 | 0.654 | 0.639 | 0.651 |
| Nv322 | 5 | 55 | 0.467 | 0.58 | 0.508 | 0.493 | 0.435 | 0.328 |
| Nv228 | 5 | 73.6 | 0.701 | 0.591 | 0.697 | 0.683 | 0.649 | 0.736 |
| Nv229 | 5 | 94.4 | 0.599 | 0.536 | 0.708 | 0.719 | 0.688 | 0.694 |
|  |  |  |  |  |  |  |  |  |
| Mean±SE |  |  | 0.53±0.03 | 0.46±0.04 | 0.58±0.04 | 0.60±0.03 | 0.56±0.03 | 0.56±0.03 |

**Table S3 Heterozygosity in HVRx generation 32 and natural *N. vitripennis* populations.** Expected heterozygosity (*H_E_*; gene diversity) per microsatellite marker. Population (sample size) and source study, and range over all natural populations. For each population the mean expected heterozygosity (± standard error) over all loci reported in this table is given.

|  | This study | Paolucci *et al.,* 2013 | | | | | | | Raychoudhury *et al.,* 2010 | |  | Grillenberger *et al.,* 2009 | | Range natural populations |
| --- | --- | --- | --- | --- | --- | --- | --- | --- | --- | --- | --- | --- | --- | --- |
| Marker | HVRx-G32 (24) | COR (18) | SWI (18) | SCH (15) | HAM (17) |  | TUR (22) | OUL (26) | NA (52) | EU (27) |  | NA (50) | EU (20) |  |
| NV109 | 0.65 |  |  |  |  | 0.78-0.92 |  |  |  |  |  | 0.78 | 0.92 | 0.78-0.92 |
| NV114 | 0.69 |  |  |  |  | 0.72-0.91 |  |  |  |  |  | 0.72 | 0.91 | 0.72-0.91 |
| NV118 | 0.66 | 0.90 | 0.91 | 0.81 | 0.85 | 0.8-0.92 | 0.86 | 0.80 |  |  |  |  |  | 0.8-0.92 |
| NV200 | 0.69 | 0.86 | 0.89 | 0.86 | 0.91 | 0.7-0.92 | 0.92 | 0.70 |  |  |  |  |  | 0.7-0.92 |
| NV205 | 0.72 | 0.95 | 0.90 | 0.91 | 0.93 | 0.79-0.95 | 0.91 | 0.79 |  |  |  |  |  | 0.79-0.95 |
| NV229 | 0.69 | 0.86 | 0.91 | 0.81 | 0.91 | 0.81-0.95 | 0.95 | 0.83 |  |  |  |  |  | 0.81-0.95 |
| NV301 | 0.28 | 0.94 | 0.93 | 0.86 | 0.84 | 0.74-0.94 | 0.94 | 0.74 |  |  |  |  |  | 0.74-0.94 |
| NV303 | 0.50 | 0.63 | 0.77 | 0.58 | 0.50 | 0.5-0.77 | 0.69 | 0.74 |  |  |  |  |  | 0.5-0.77 |
| Nv319 | 0.65 | 0.88 | 0.91 | 0.79 | 0.92 | 0.79-0.95 | 0.95 | 0.87 |  |  |  |  |  | 0.79-0.95 |
| NV300 | 0.16 |  |  |  |  | 0.46-0.55 |  |  | 0.51 | 0.47 |  | 0.55 | 0.46 | 0.46-0.55 |
| NV303 | 0.50 |  |  |  |  | 0.73-0.76 |  |  | 0.76 | 0.73 |  |  |  | 0.73-0.76 |
| NV308 | 0.08 |  |  |  |  | 0.64-0.8 |  |  |  |  |  | 0.64 | 0.80 | 0.64-0.8 |
|  |  |  |  |  |  |  |  |  |  |  |  |  |  |  |
| Mean±SE | 0.52±0.07 | 0.86±0.04 | 0.89±0.02 | 0.80±0.04 | 0.84±0.06 | 0.83±0.05 | 0.89±0.04 | 0.78±0.02 | 0.64±0.1 | 0.60±0.1 |  | 0.67±0.05 | 0.77±0.11 |  |
|  |  |  |  |  |  |  |  |  |  |  |  |  |  |  |
